# Analytics-statistics mixed training and its fitness to semisupervised manufacturing

**DOI:** 10.1371/journal.pone.0220607

**Published:** 2019-08-13

**Authors:** Parag Parashar, Chun Han Chen, Chandni Akbar, Sze Ming Fu, Tejender S. Rawat, Sparsh Pratik, Rajat Butola, Shih Han Chen, Albert S. Lin

**Affiliations:** 1 College of Electrical Engineering and Computer Science, National Chiao-Tung University, Hsinchu, Taiwan; 2 Institute of Electronics Engineering, National Chiao-Tung University, Hsinchu, Taiwan; Newcastle University, UNITED KINGDOM

## Abstract

While there have been many studies using machine learning (ML) algorithms to predict process outcomes and device performance in semiconductor manufacturing, the extensively developed technology computer-aided design (TCAD) physical models should play a more significant role in conjunction with ML. While TCAD models have been effective in predicting the trends of experiments, a machine learning statistical model is more capable of predicting the anomalous effects that can be dependent on the chambers, machines, fabrication environment, and specific layouts. In this paper, we use an analytics-statistics mixed training (ASMT) approach using TCAD. Under this method, the TCAD models are incorporated into the machine learning training procedure. The mixed dataset with the experimental and TCAD results improved the prediction in terms of accuracy. With the application of ASMT to the BOSCH process, we show that the mean square error (MSE) can be effectively decreased when the analytics-statistics mixed training (ASMT) scheme is used instead of the classic neural network (NN) used in the baseline study. In this method, statistical induction and analytical deduction can be combined to increase the prediction accuracy of future intelligent semiconductor manufacturing.

## 1. Introduction

**Fig 1:**
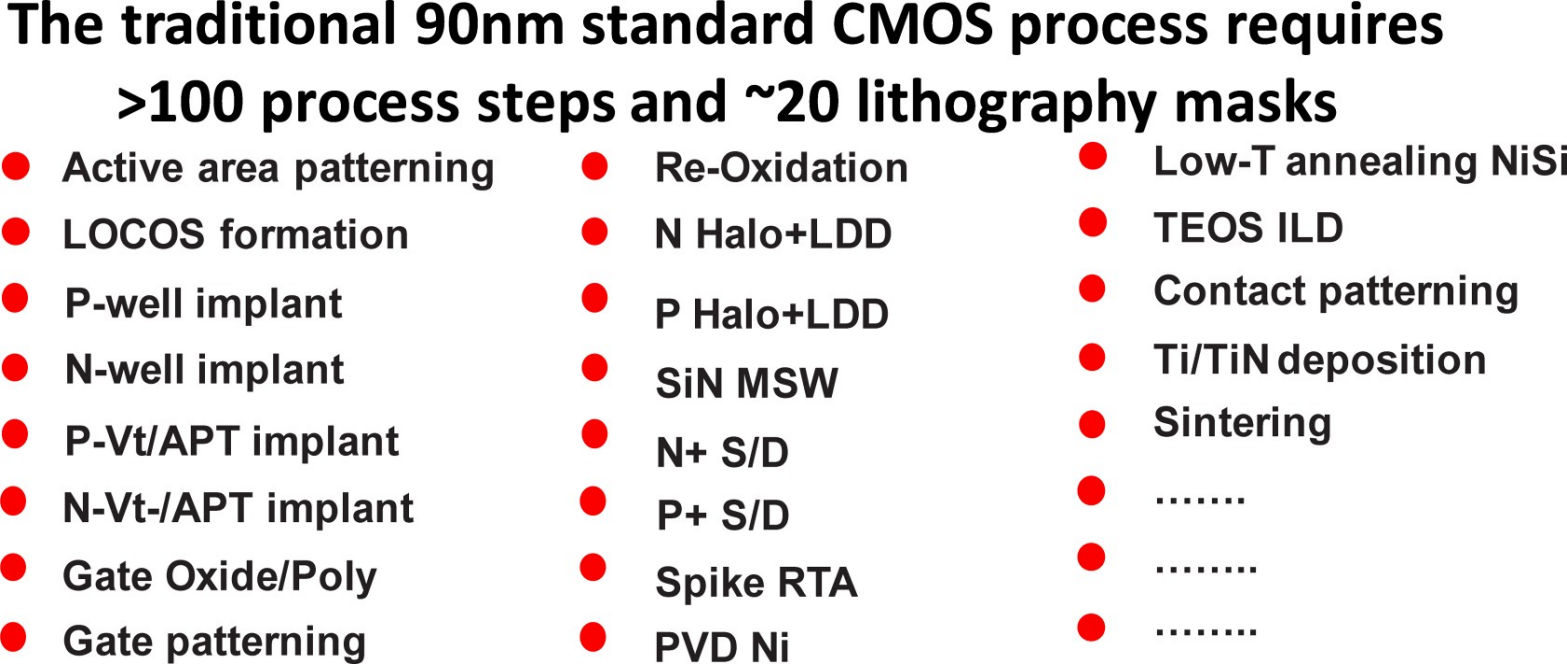
The traditional 90 nm standard CMOS process. This process requires more than one hundred steps. There can be several hundred parameters in the fabrication flow. Abbreviations: Low-T—low temperature, LOCOS- local oxidation of silicon, Halo+LDD—halo-type lightly doped drain, TEOS ILD-tetraethoxysilane interlayer dielectric, MSW- major sidewall spacers, Vt/APT- threshold voltage for anti-punch through, poly- polysilicon, S/D- source/drain, RTA- rapid thermal processing, PVD- physical layer deposition, and N+/P+—n-type /p-type dopants.

Currently, machine learning is widely applied to many fields, such as medical imaging [1,2,3,4], financial crises [5,6], biology [7,8,9,10,11], and traffic classification [12,13,14]. Machine learning is utilized to predict the results of future experiments under various conditions by incorporating a small amount of known or experimental data for training. Semiconductor manufacturing is a complicated process that requires the monitoring of many of parameters during the process steps such as deposition, lithography, and etching, as depicted in **Fig 1**. A large number of input features can exist at a single instant of time during the process cycle. Thus, the complexity of the irregular sample space together with the large number of input features makes predicting the semiconductor process a very challenging problem. This problem paves way for the use of machine learning in this area to find optimal solutions. There have been some previous studies on machine learning applied to semiconductor manufacturing [15,16,17,18,19,20,21,22,23,24,25,26]. Duo Ding et al.[27] used machine learning to detect lithography hotspots on wafers. Park and Shin [28] tried to solve the issue of field management in semiconductor manufacturing with the help of machine learning. Kim et al. [29] proposed a generalized regression neural network (GRNN) in plasmon dry etching. All these efforts have provided great insights into the possible implementation of machine learning in semiconductor manufacturing processes. One important issue in the case of using machine learning for smart manufacturing is that to achieve increased model accuracy, more training data are required. Nonetheless, more data mean a higher cost in the trial-and-error stages, and this will further increase the cost of the expensive semiconductor flow cycles.

To accelerate the machine learning model construction and prediction within a reasonable number of experimental runs, we use an analytics-statistics mixed trained (ASMT) neural network (NN) using technology computer-aided design (TCAD). This kind of constraint-based learning has been successful in many fields [30,31,32,33,34]. The constraints can be derived from physics, chemistry, human sense, or a small pre-trained dataset to provide uniform constraints over a larger or multiple datasets. Different from the traditional supervised neural network that uses only experimentally labeled data in the training process, analytics-statistics mixed training (ASMT) uses both the experimentally labeled and TCAD-labeled data to increase the model prediction accuracy or decrease the required amount of experimentally labeled data to reduce the cost. Additionally, the methodology of using statistics and analytics jointly can easily fit semisupervised learning (SSL) framework [31]. While there have been many past efforts in smart semiconductor manufacturing, in this paper, we use the TCAD simulation results to label the unlabeled data by SSL, which has been thoroughly investigated in this field. The application of TCAD-labeled analytics-statistics mixed training (ASMT) to semisupervised learning (SSL) will be explained in the following paragraph.

Supervised learning constructs an input-output relationship with trained statistical models and labeled data. With the help of SSL, the unlabeled data can effectively support the statistical model prediction in some cases if an appropriate algorithm is used to incorporate the information provided by the unlabeled data into the model. There has been much literature to date showing that adding unlabeled data into the training procedure still improves the prediction accuracy of statistical models even when the output is missing [35]. Semisupervised learning can be implemented with various algorithms, such as self-training (ST), expectation maximization (EM), transductive support vector machine (TSVM), cotraining, multiview, and graph-based semisupervised learning. The self-training (ST) algorithm is used when the labeled dataset has a smaller amount of data than the unlabeled dataset. It consists of a few steps, including training, prediction, selection of proper labels for the unlabeled data, retraining an enlarged labeled dataset, and continuation until convergence is achieved. ST is the earliest semisupervised learning algorithm, in which the unlabeled data are gradually assigned a label (output) during the training procedure [36]. The expectation-maximization (EM) algorithm assumes initial values for the labels of unlabeled data. The prediction is made after training, and the convergence is defined as the predicted labels being consistent with or within an error tolerance compared to the assumed labels [5]. In the case of a transductive support vector machine (TSVM), maximizing the decision boundary margin in the input vector space is the ultimate goal when labeling the unlabeled data. [37].

The TCAD-labeled analytics-statistics mixed training (ASMT) scheme can be used with SSL in the sense that the unlabeled data in SSL can be labeled using analytical models, if they exist, to enhance the accuracy of SSL. Specifically, the TCAD analytical model values can be used as the initial guess in the expectation-maximization (EM) algorithm, or serve as the discriminant to assist in the selection procedure to merge the likely to-be-true unlabeled data into the labeled group.

**Fig 2:**
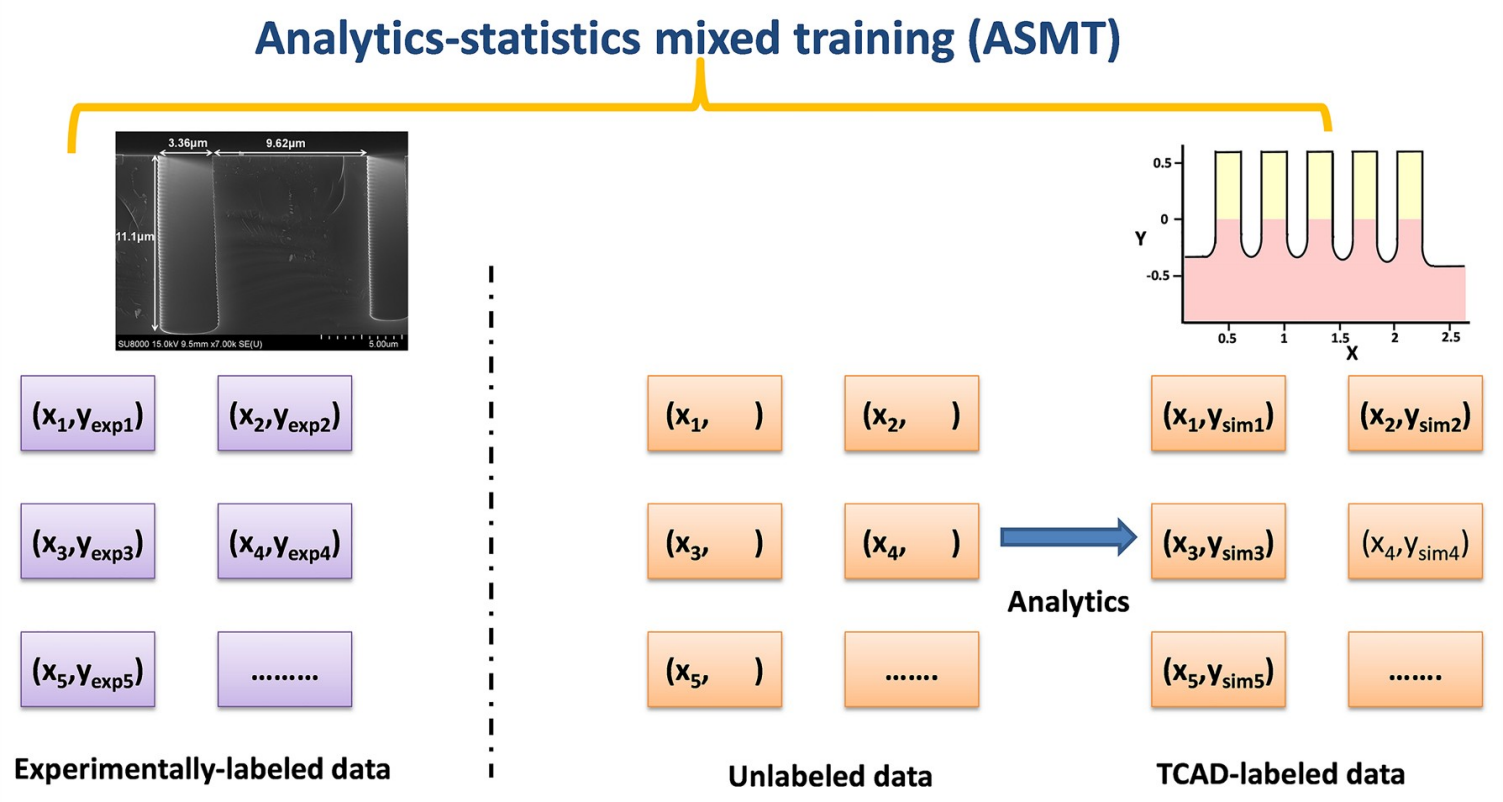
TCAD-labeled analytics-statistics mixed training (ASMT) uses the experimentally labeled and analytically labeled data in conjunction with the TCAD simulations. The model fits the into the SSL framework well since the unlabeled data can be labeled in SSL using the analytical models and then be fed into supervised learning models. The analytics-labeled data can also serve as the initial guess in the expectation-maximization algorithm under SSL or the selection criteria in self-training.

Combining physics, chemistry, or biology with machine learning methods have been drawing increased attention in recent years [30,31]. Here, we use an approach using the TCAD simulations so that the model can be trained using statistics and analytics. The idea is shown in Fig 2. Using the deep reactive ion etching (DRIE) Bosch process [38,39,40] as an example, Fig 2 illustrates the concepts of our model. In this model, we utilized the experimental and TCAD data simultaneously to attain accurate and faster predictions. First, we build a TCAD model to predict the etching depth (*d*) with various input features: the pattern width (*W*), the etching time (*t*), trench space (*S*), pressure main etch (*P*), SF_6_ flow main etch (*F*) and inductively coupled plasma (ICP) RF power in main etch (*IF*). During the preprocessing of the input data, space (*S*) is eliminated because we are using a constant space value in our experiment. The other 5 input parameters are critical in determining the etching depths and are considered to be independent. The etched patterns are designed with different line widths and spacing. After the TCAD calculations, the TCAD results are used to label the unlabeled data. Thereafter, both the experimentally labeled and TCAD-labeled data are supplied to the NN for optimal training.

## 2. Methods

### 2.1 Analytics-Statistics Mixed Training (ASMT)

The first paper regarding including physical models into semi-supervised learning is by Ren et. al[31]. The basic idea in this work is to regard the analytical technology computer-aided design (TCAD) outputs as the target output values. Here, we used the aspect-ratio dependent etching (ARDE) process to demonstrate the effectiveness of the TCAD-based analytics-statistics mixed training (ASMT). Although the TCAD model is not entirely accurate, especially in our case, we use a very simple analytical model to calculate the ARDE effect. The inclusion of this additional unlabeled data still helps to improve the accuracy of the neural networks. This improvement will be evident when the test set prediction is made.

**Fig 3:**
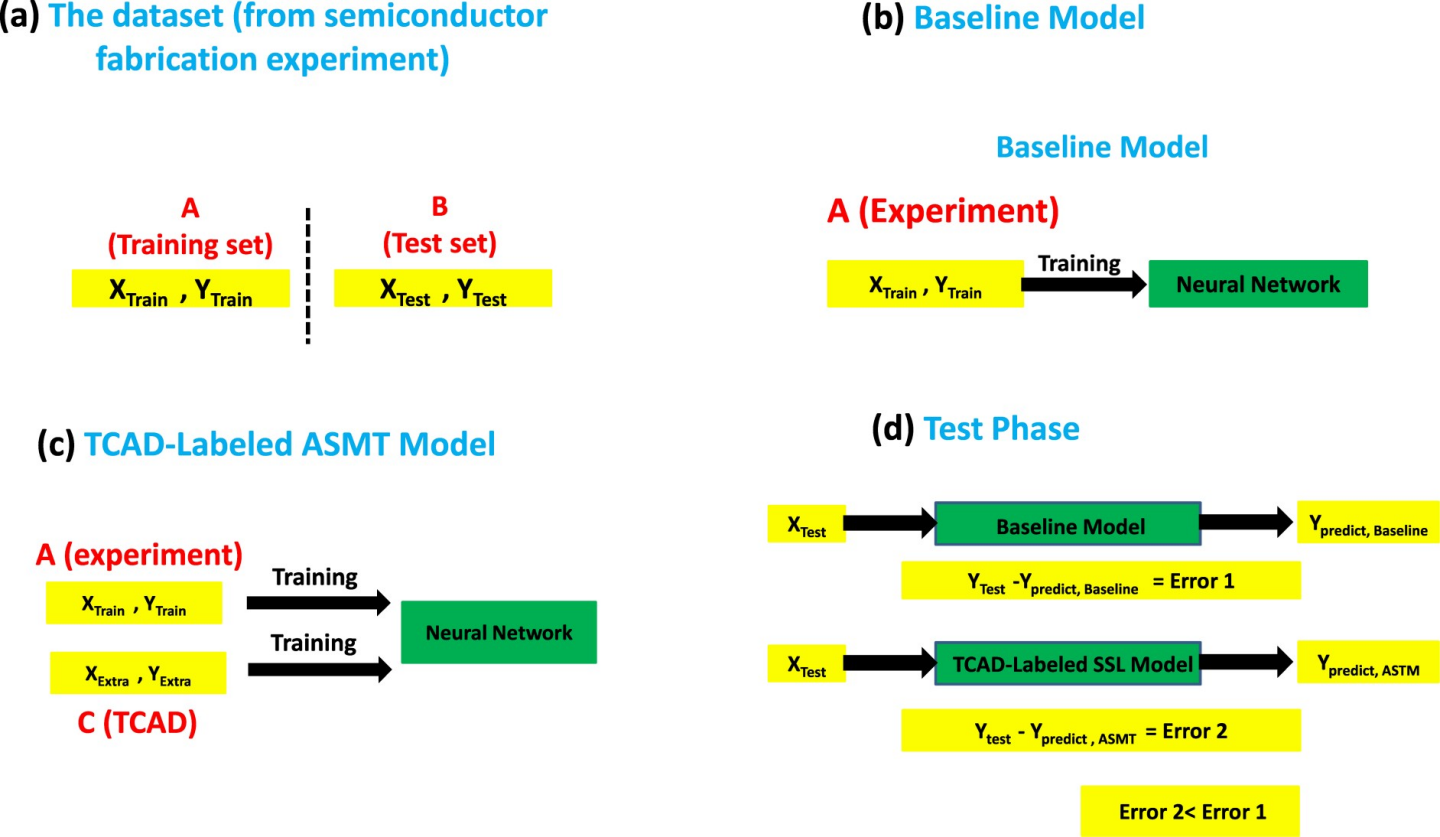
Implementation of TCAD-labeled ASMT. (a) The dataset is split into the training set and test set. (b) The baseline model is trained with the experimentally labeled data. (c) The TCAD-labeled ASMT model is trained with the experimentally labeled and TCAD-labeled data. (d) The test phase of the baseline and TCAD-labeled ASMT models.

The implementation of our work is demonstrated in Fig 3. The set of the experimentally labeled data was divided into a training set (X_Train_, Y_Train_) and a test set (X_Test_, Y_Test_), and the boundary is denoted by the *partition* parameter in this paper. The test set is reserved for testing the prediction accuracy of the baseline and TCAD-labeled ASMT models. The baseline NN model is trained with the experimentally labeled data in the training set. For the ASMT model, the TCAD-labeled data, (X_Extra_, Y_Extra_), can be added to the training set. Therefore, the extended training set with the experimentally labeled and analytically labeled data is expected to provide a better prediction accuracy. It is worth mentioning how to select the input parameter values, X_Extra_, for the TCAD-labeled data to form the extended training set. In fact, the selection can depend highly on the machine learning problem under investigation. In the case of smart manufacturing, we can determine the input parameters that will be tried for future experiments, and therefore, the selection of the input parameter values can be done in the same way as the values in the test set. The same situation exists for algorithmic trading, where the input parameter values, such as the historic high/low price and volume history, can be known in advance. In some machine learning problems, such as natural language processing or sentiment analyses, the future input parameter values may not be known in advance, and in this case, the selection of the input parameter values in the ASMT can be made in as wide a range as possible to cover the potential input parameter values in future experiments or equivalently in the test set. We use maximum likelihood (MLE) inference during model training, and we can locate the *w*_ij_ values of the NN using the stochastic gradient descent (SGD) method.

### 2.2 Neural network (NN) structure

A neural network (NN) [41] is considered to be a computational model that produces output data from input data and corresponding weights, *w*_ij_, using a nonlinear activation function. Usually, monotonically increasing functions are used as activation functions in this context. The purpose of using monotonically increasing activation functions is to generate a generalized model for any complex interaction. Data preprocessing is considered an important step before training the NN model and is done by normalizing the input data into a consistent range of 0 to 1 so that the SGD method converges more easily. The normalization is achieved by the min-max scalar. Different from conventional machine learning methods, here, we have two datasets. The experimental dataset consists of the fabrication data, which are divided into the training set (X_Train_, Y_Train_) and the test set (X_Test_, Y_Test_), and the TCAD dataset (X_Extra_, Y_Extra_) from the analytical modeling, as illustrated in Fig 3. The normalization of the TCAD dataset is performed simply by extracting the minimum and maximum of the dataset since the TCAD values are known in advance. In extracting of the minimum and maximum of the experimental dataset, the estimate is made by multiplying the TCAD dataset minimum and maximum by the ratio of the experimental etching depth value to the TCAD calculation value of the first collected experiment data sample. When we denormalize the normalized etching depth values back to the real etching depth values, for the prediction or mixed experiment-TCAD data during training, the denormalization is uniformly based on the min-max scalar derived from fitting the experimental dataset, as will be shown in section 3.

The ReLU activation function has better convergence than the sigmoid and tanh activation functions. Specifically, the cutoff at x<0 and the linearity at x≥0 ensure a simple gradient formulation, which prevents exploding gradient values and subsequent divergence during training.

**Fig 4:**
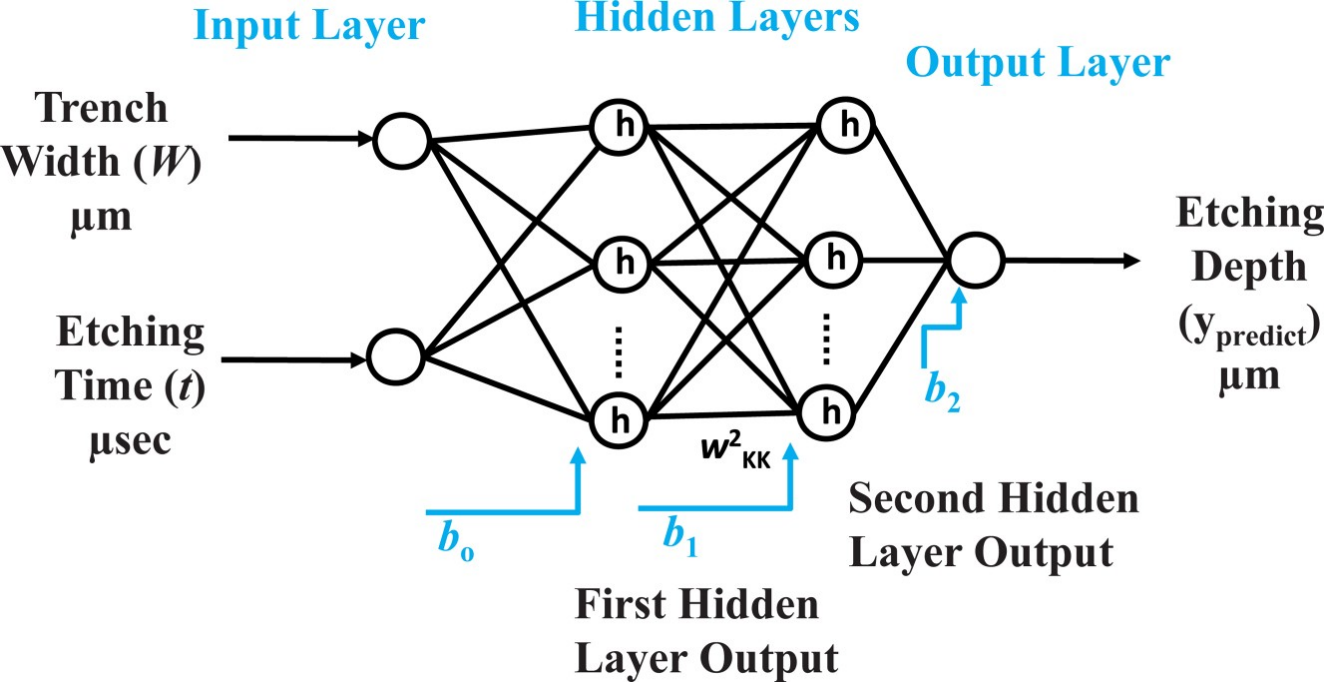
General structure of a four-layer NN with 2 input features, two hidden layers with 50 neurons in each layer, and one output layer.

Consider an NN with an n-dimensional input vector with the input parameters *X* = {*x*_1_, *x*_2_,*…*, *x*_n_}. It has two hidden layers with 50 neurons in each layer, and the ReLU activation function is represented as *h* as shown in Fig 4. The output, $g_{i}^{1}$, of the first hidden layer from any arbitrary neuron, *I*, is given as:

$$g_{i}^{1}=\sum_{j=1}^{p}h(w_{\mathrm{ij}}^{1}\times x_{j}+b_{0})$$ (1)

where *b*_0_ is the bias in the first hidden layer, *p* is the total number of input data samples, $w_{\mathrm{ij}}^{1}$ is the weight between the *j*^th^ node in the input layer and *i*^th^ neuron in the hidden layer. *x*_j_ is the input vector of the *j*^th^ feature.

The ReLu activation function used in this work since our dataset has positive real values, and we need the etching depth to be a positive value in micrometers. The ReLU activation function is

$$h(x)=\max(0,x)=\{\begin{array}{l} x,\;if\;x\geq 0\\ 0,\;\mathrm{Otherwise} \end{array}$$ (2)

To calculate the output from the second hidden layer, the output of the first hidden layer is considered as its input. Therefore, the output of any *s* neurons in the second hidden layer, $g_{s}^{2}$, can be generalized as:

$$g_{s}^{2}=h(\sum_{i=1}^{k}g_{i}^{1}\times w_{\mathrm{is}}^{2}+b_{1})$$ (3)

where *b*_1_ is the bias for the second hidden layer, *g*^2^_,_ and *k* is the total number of neurons in the first hidden layer.

The final output is the etching depth, which can be computed by summing all of the outputs from the second hidden layer. The mathematical notation for the output, *y*_predict_, is given as

$$y_{\mathrm{predict}}=\sum_{j=1}^{k}g_{j}^{2}\times w_{j1}^{{}_{3}}+b_{2}$$ (4)

where *b*_*2*_ is the bias, $w_{j1}^{{}_{3}}$ represents the weights, *k* is the total number of neurons, and *y*_predict_ is the predicted etching depth.

**Fig 5:**
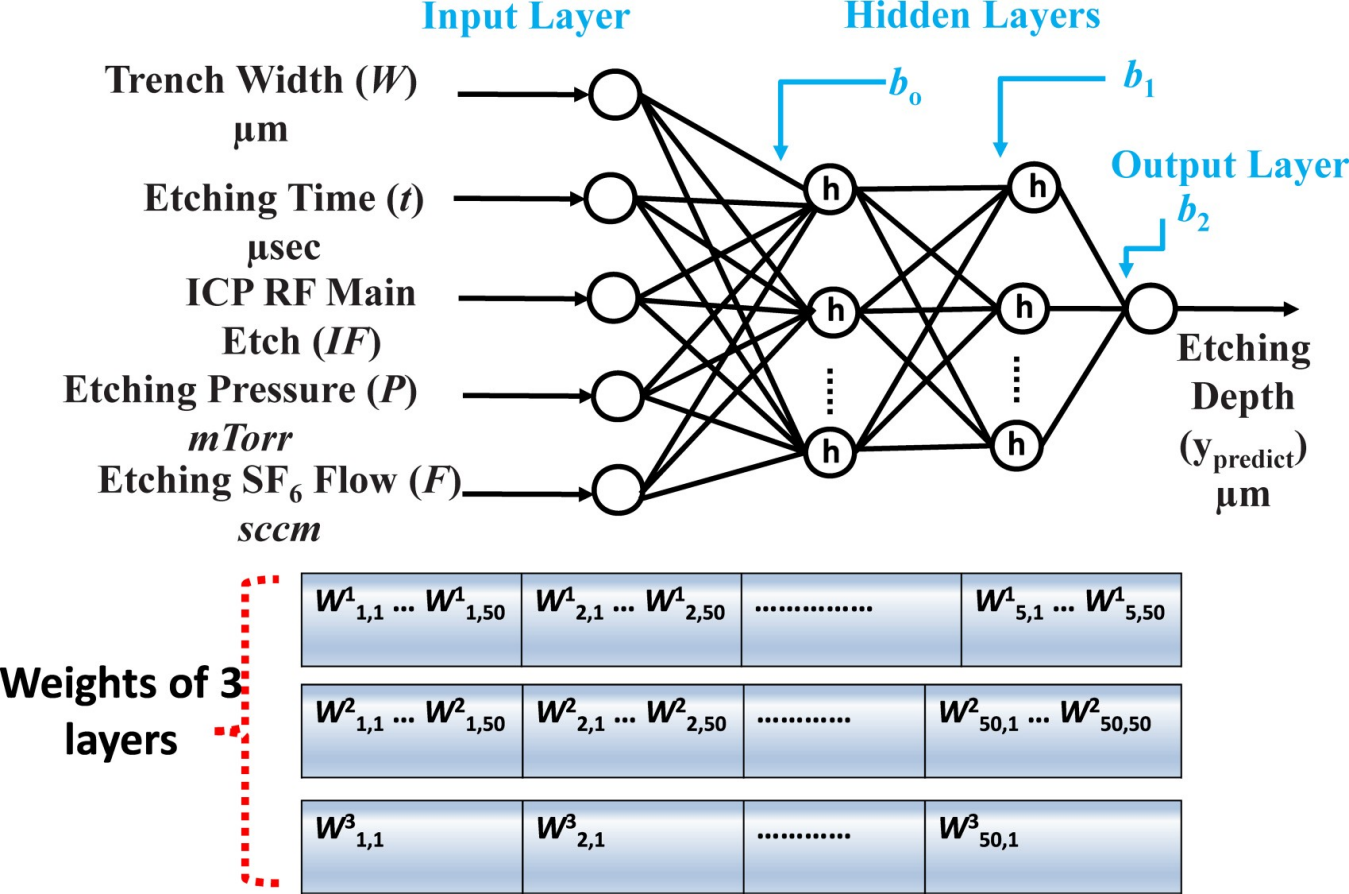
General structure of a four-layer NN with 5 input features, two hidden layers with 50 neurons in each layer, and one output layer.

In this paper, we have implemented the NN using a multilayer perceptron (MLP) by using 2-input and 5-input datasets. The corresponding features are illustrated in Figs 4 and 5, respectively.

#### 2.2.1 The NN loss function in a multilayer perceptron (MLP)

In machine learning, the loss function must be defined before the training procedure. The loss function is expected to be minimized if convergence is observed. In the case of our regression problem, we have used a squared error loss function. For the baseline algorithm, the experimental data were used to train the NN model. The mathematical expression of the loss function for the baseline training set is given as

$$loss(y_{\mathrm{Train}},w)=\frac{1}{2}{\left\|y_{\mathrm{Train}}-y_{\mathrm{predict}}\right\|}^{2}+\frac{\alpha }{2}{\left\|w\right\|}^{2}$$ (5A)

where *y*_Train_ represents the experimentally labeled data, and *w* represents the corresponding weights. For complex models, α||*w||*^*2*^ is the regularization term, where α cannot be negative, and its value is 0.001 in our model. The predicted value of etching depth is *y*_predict_.

As explained earlier, the TCAD-labeled data are added to the experimental dataset to better train the NN model. The loss function for TCAD-labeled ASMT is given as

$$loss(y_{\mathrm{Train}},y_{\mathrm{Extra}},w)=\frac{1}{2}{\left\|y_{\mathrm{Train}}-y_{\mathrm{predict}}\right\|}^{2}+\frac{1}{2}{\left\|y_{\mathrm{Extra}}-y_{\mathrm{predict}}\right\|}^{2}+\frac{\alpha }{2}{\left\|w\right\|}^{2}$$ (5B)

where α||*w||*^*2*^ is the regularization term, *d* is the predicted value of the etching depth, *y*_Train_ and *y*_Extra_ are the experimentally labeled and TCAD-labeled values, respectively.

#### 2.2.2 The Adam optimizer

The minimization of the loss function is achieved with appropriate solvers or optimizers. From the Scikit-learn library for the Python language [42,43], we utilized the Adam optimizer [44]. While it shares some similarity to the stochastic gradient descent method, the Adam optimizer uses adaptive estimates and takes into account only the lower-order moments during model parameter tuning. The neural network weights are updated in the Adam optimizer as

$$w^{i+1}=w^{i}-\eta (\alpha \frac{\partial h(w)}{\partial w}+\frac{\partial Loss}{\partial w})$$ (6)

where *w*^i+1^ and *w*^i^ are the updated and previous weights, *ƞ* is the learning rate, ∂*h*(*w*)/ ∂*w* and ∂*Loss*/∂*w* are the derivatives of the activation function and the loss function with respect to the weight, respectively.

For the sake of converged solutions, the optimizer, learning rate, activation function and tolerance all play vital roles. Our model possesses an MLP regressor and a ReLU activation function with a tolerance of 0.00001.

#### 2.2.3 The mean square error (MSE)

The mean squared error (MSE) metric is used to assess the fitting or prediction accuracy in our case. The predicted output, *y*_predict,_ is compared to the corresponding target values in the training and test datasets. The mathematical forms of the MSE are given below for the training set. The MSE formulas are somewhat different for the baseline model using a conventional NN and for TCAD ASMT:

$$MSE_{\mathrm{baseline}}=\frac{1}{2}{\left\|y_{\mathrm{Train}}-y_{\mathrm{predict}}\right\|}^{2}$$ (7A)

$$MSE_{\mathrm{TCAD}\;\mathrm{ASMT}}=\frac{1}{2}{\left\|y_{\mathrm{Train}}-y_{\mathrm{predict}}\right\|}^{2}+\frac{1}{2}{\left\|y_{\mathrm{Extra}}-y_{\mathrm{predict}}\right\|}^{2}$$ (7B)

The mathematical formulation of the MSE for the test set is uniformly defined as the difference between the predicted depth (*y*_predict_) and the true experimental depth in the test set,

$$MSE=\frac{1}{2}{\left\|y_{\mathrm{Test}}-y_{\mathrm{predict}}\right\|}^{2}$$ (7C)

### 2.3 The TCAD model and BOSCH DRIE etching

A six inch (100) p-type B-doped Si wafer is used for this experiment. An automated spin-coater, TEL CLEAN TRACK MK-8, and Leica Weprint 200 E-beam stepper are used for e-beam resist patterning. There are two sets of experiments involving the DRIE Bosch process [45] in this work. The first dataset [46] initially had three inputs, the etching time (*t*), trench width (*W*) and trench space (*S*). However, during preprocessing, the trench space (*S*) is excluded from the input parameters due to its small variations which have no effect on the etching depth. The three input parameters are listed in Table 1 along with their corresponding values and units.

**Table 1:** The input features for the aspect-ratio dependent etching (ARDE) experiments for the 2-input dataset [46].

Parameters	Value	Units
**Space**	5, 1, 0.5	μm
**Etching time**	170, 68, 51, 34	μsec
**Line width**	1, 0.8, 0.5, 0.3	μm

In the second dataset, we originally had six input features. However, the trench space (*S*) is eliminated due to the reason mentioned above. The variations of features in the 5-input dataset are listed in Table 2.

**Table 2:** The input features for the aspect-ratio dependent etching (ARDE) experiments for the 5-input dataset set.

Parameters	Value	Units
**Pressure**	20, 40, 60	mTorr
**Trench Width**	4.9~0.3	μm
**Trench Space**	10	μm
**ICP RF power**	1000, 1500, 1250	W
**SF**_**6**_ **flow**	150, 200, 250	sccm

**Fig 6:**
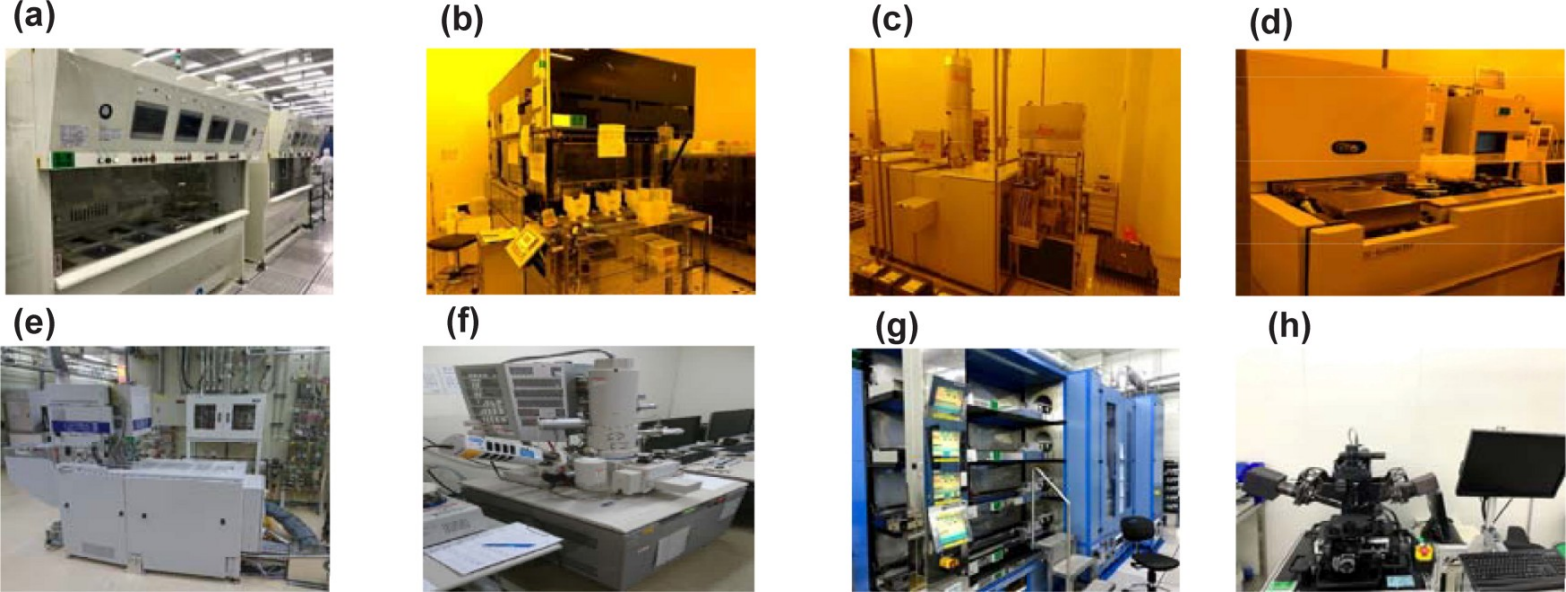
Machines Used. (a) Wet bench (b)Track (c) Leica E-beam (d) In-line SEM (e) Deep RIE (f) SEM (g) Furnace (h) Ellipsometry.

The entire process flow of the machines used in this work is shown in Fig 6. In our BOSCH DRIE process using the Oxford^TM^ machine, the microloading is not very pronounced because of our one-dimensional (1D) etch patterns. As a result, the spacing between adjacent trenches is not taken into account in the machine learning models. If dense-pattern two-dimensional (2D) etching is conducted, the microloading will be more pronounced. Nevertheless, we want to emphasize that the same TCAD-labeled ASMT learning scheme can be easily employed to predict the 2D etch by taking into account the spacing and other necessary input features.

**Fig 7:**
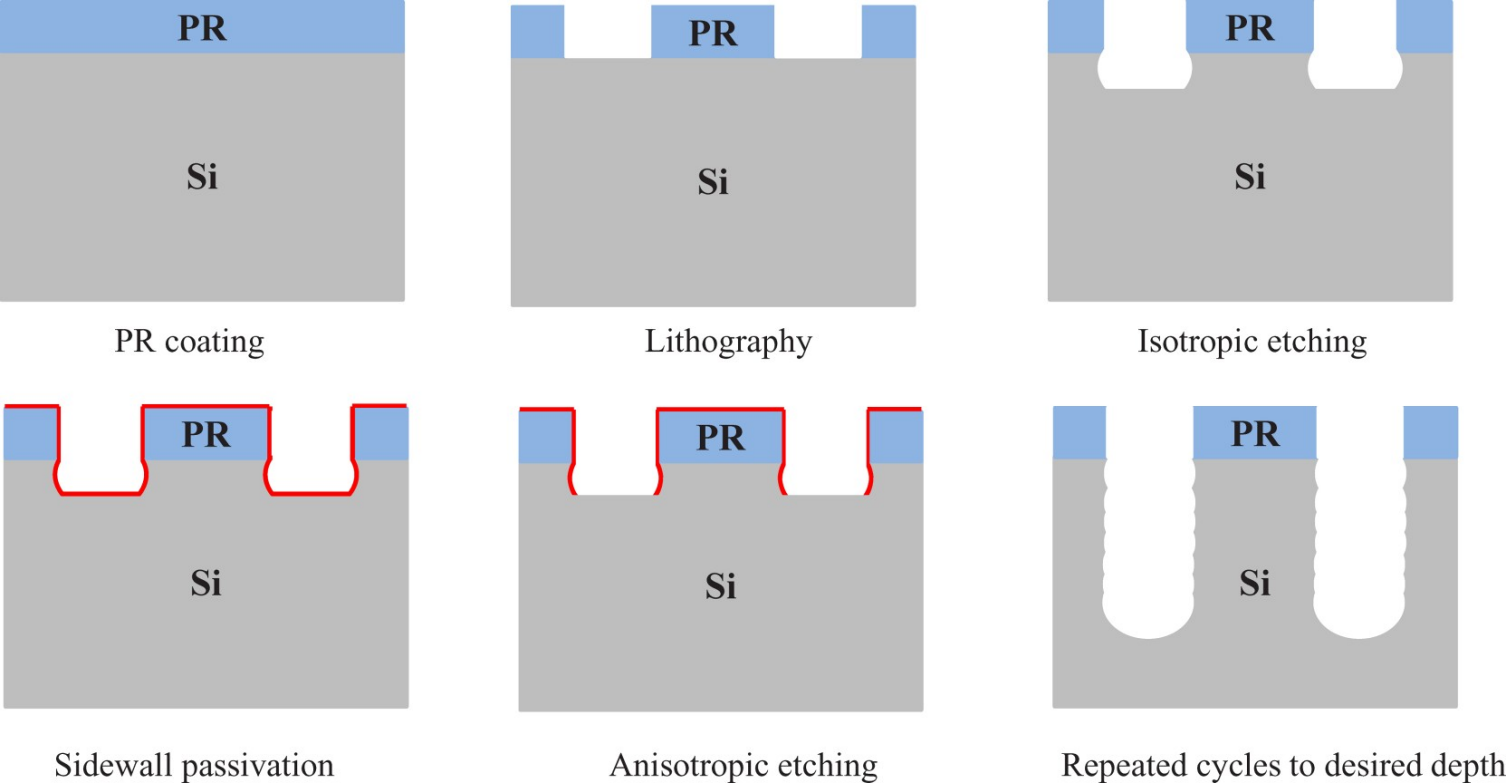
Illustration of the trenching in the Bosch etching process [45,47].

**Fig 8:**
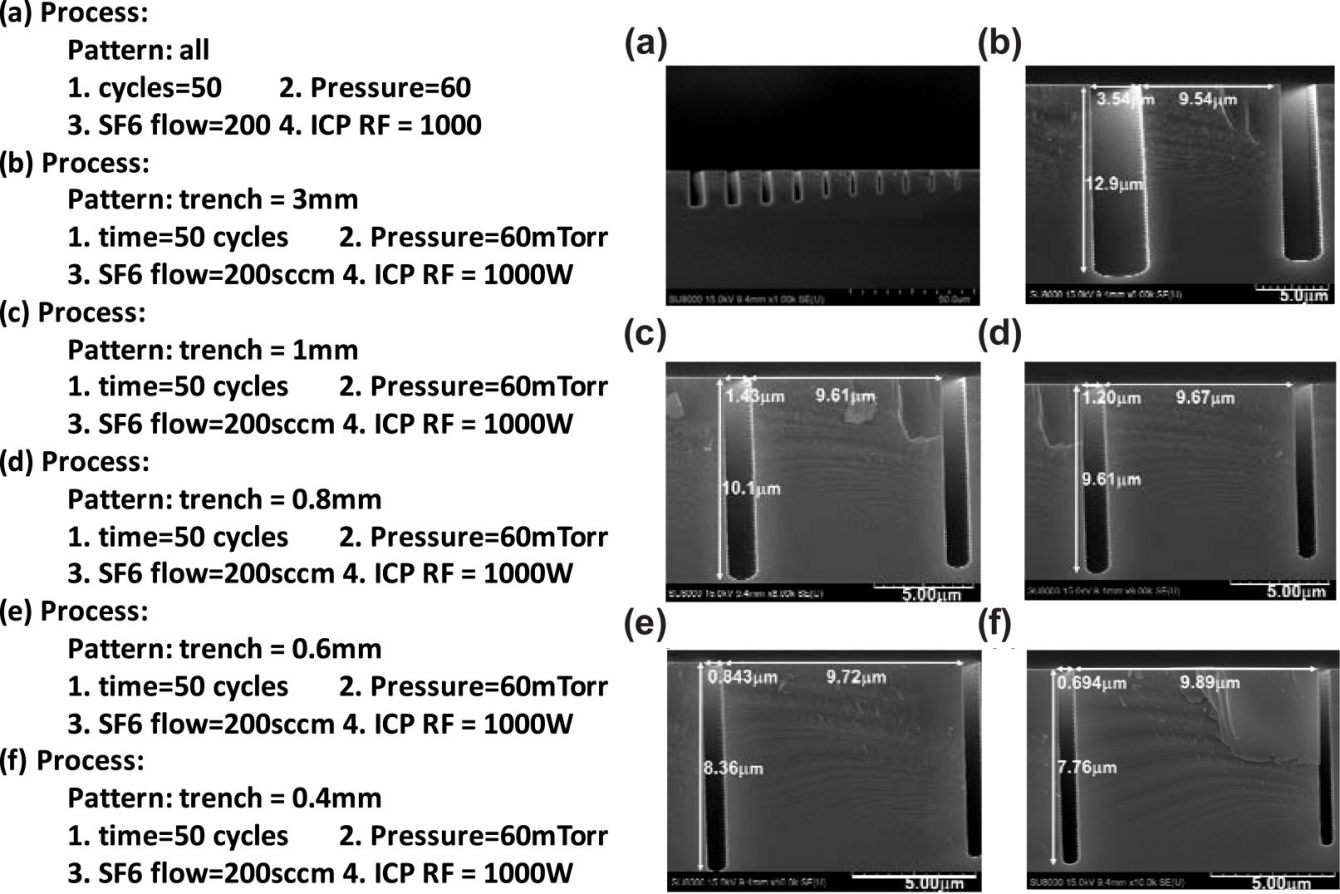
Scanning electron microscope images for different process conditions.

The Bosch process [45] for Si DRIE is conducted using the Oxford^TM^ Estrelas 100 inductively coupled Plasmon (ICP) reactive ion etching (RIE) machine. Fig 7 illustrates the Bosch etching process steps. A Hitachi SU-8010 scanning electron microscope (SEM) was used to examine the etching line widths and depths for the etched trenches. A wavy sidewall, which is a characteristic of the Bosch DRIE process, is evident in our SEM micrographs shown in Fig 8.

The etching depth, *d* = *y*_TCAD_, is computed by considering the gas flow conservation equation [38]

$$v_{t}-(1-k(\frac{d(t)}{W}))v_{t}-k(\frac{d(t)}{W})(1-s)v_{b}=sv_{b}.$$ (8)

The ARDE effect will be reflected in Eq 8. The transmission probability or Knudsen coefficient is *k*; *v*_t_ is the flux incidence of gas at the top of the etched feature; *v*_b_ is the flux species at the bottom of the trench; *s* is the reaction probability at the trench bottom; *d*(*t*) is the trench depth at time *t*; and *W* is the trench width. The full model description can be referred to in the IBM paper. The final etching depth predicted by TCAD is computed by numerical integration,

$$\begin{array}{l} d(t=t_{end})=\int_{t=0}^{t=t_{end}}R(\frac{d(t)}{W})dt\\ \;=\int_{t=0}^{t=t_{end}}R(0)\frac{k(\frac{d(t)}{W})}{k(\frac{d(t)}{W})+(1-k(\frac{d(t)}{W}))s}dt \end{array}$$ (9)

*R*(0) is the etching rate at the top of the trench; *R(d*(*t*) */ W)* is the etching rate at the bottom of the trench; *t*_end_ is the time when the Bosch process ends.

## 3. Results and discussion

**Fig 9:**
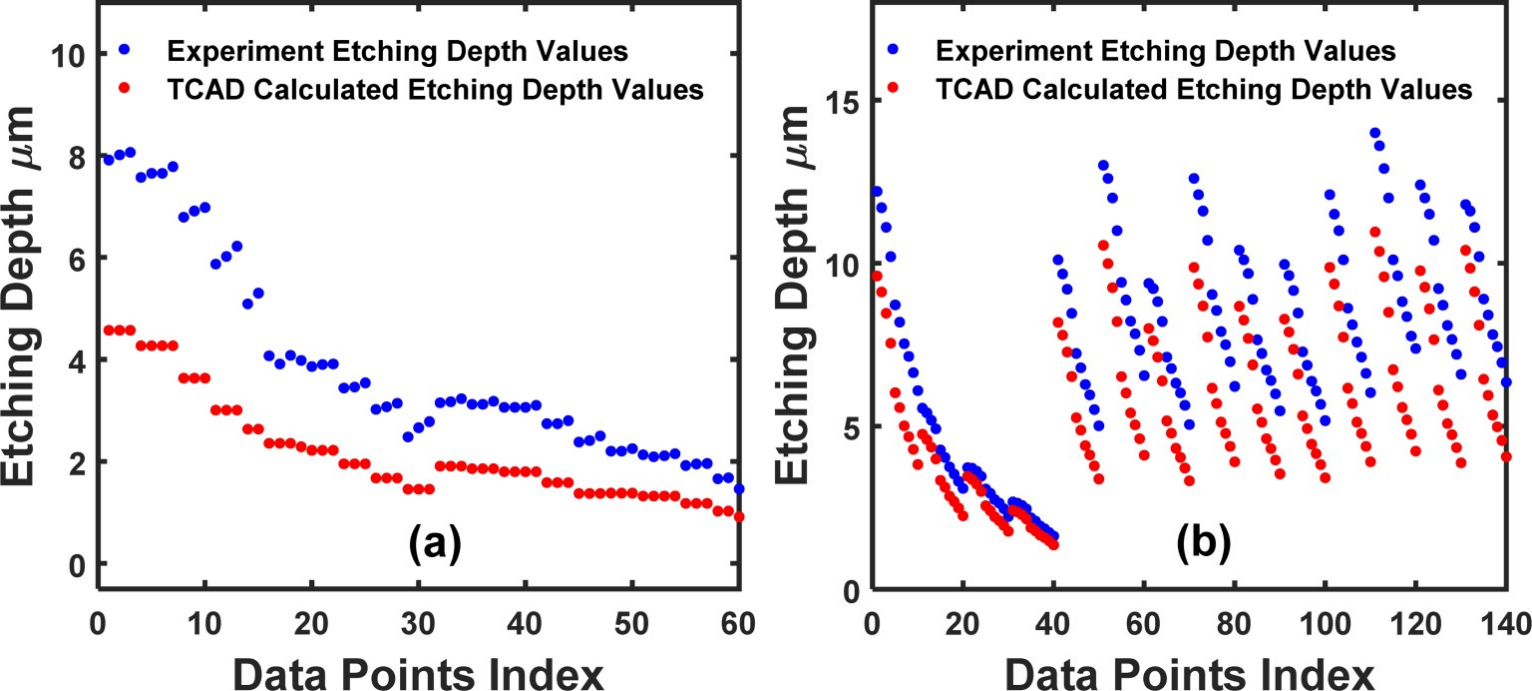
Datasets. (a) 2-input dataset; (b) 5-input dataset; both the experimental and TCAD datasets are shown.

In this section, the *partition* parameter assumes values of 15, 30 and 45 for the 2-input dataset and 10, 20, 30, 40 for the 5-input dataset. After partitioning, a baseline NN is trained with the training set of the experimental dataset (X_Train_, Y_Train_), while the amount of experimental data to be trained depends on the *partition* parameter. For the ASMT proposal, TCAD is used to supplement machine learning by supplying the additional data (X_Extra_, Y_Extra_) with the TCAD calculated outputs/labels. An ASMT NN is trained with the experimental data (X_Train_, Y_Train_) plus the TCAD-labeled data (X_Extra_, Y_Extra_), which is our TCAD-assisted ASMT model. Afterward, the test dataset from the experiment (X_Test_, Y_Test_) are used to verify the prediction accuracy of the baseline and TCAD-assisted ASMT models. The detailed model description, dataset annotation, and normalization and denormalization procedures are clearly described in sections 2.1 and 2.2. Fig 9 shows the experimental and TCAD datasets in this work.

**Fig 10:**
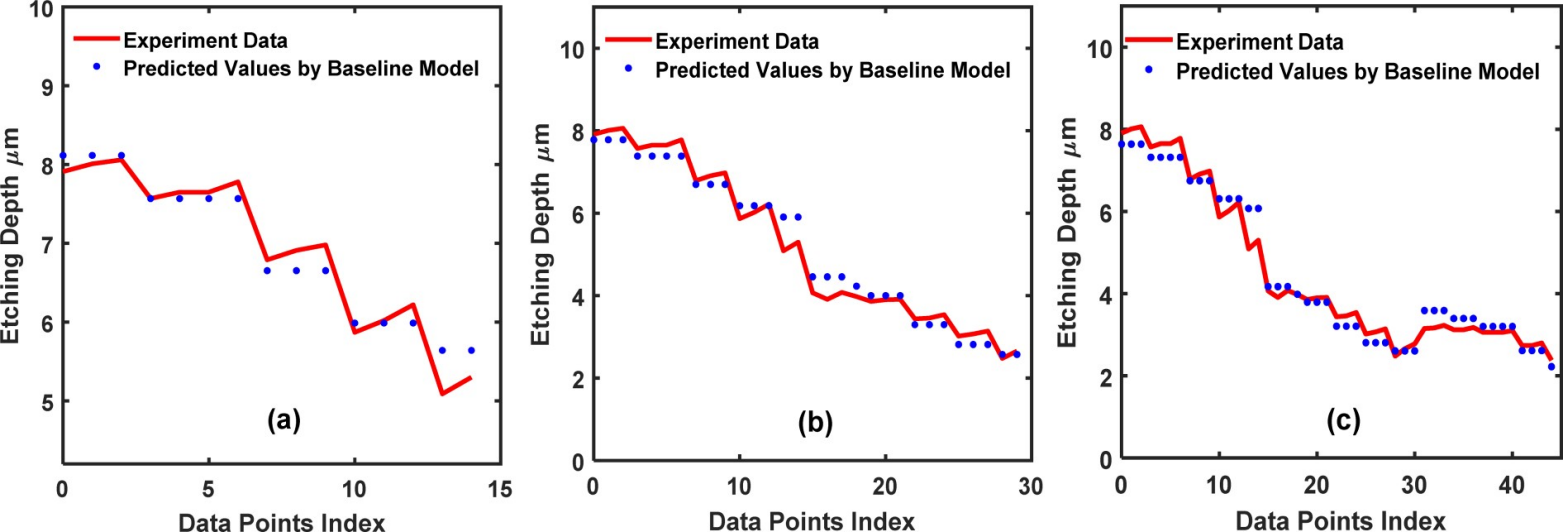
Training dataset fitting for the 2-input dataset using the baseline conventional NN. The partition values, 15, 30, and 45, are shown in (a), (b) and (c), respectively.

In Fig 10(A), we show the results of the training set fitted by the baseline neural network using *partition* = 15. There are 2 hidden layers with 50 neurons per hidden layer. The fit for the training set is satisfactory with an MSE = 1.3×10^−3,^ as shown in Table **3**. Rather than using only the experimental data, our TCAD-assisted analytics-statistics mixed training (ASMT) model takes both the experimental and TCAD-labeled data for training to illustrate the effectiveness of TCAD-assisted ASMT. With the help of the TCAD calculations, the effectiveness of ASMT learning becomes very pronounced. For the TCAD-labeled data (X_Extra_, Y_Extra_), the TCAD value, Y_Extra_, is calculated by the model in Eq 9.

**Table 3:** MSE values of the training and testing of the 2-input dataset for the TCAD-labeled ASMT algorithm.

Partition value	15	30	45
**Baseline training**	0.001324693	0.002322580	0.002385333
**Baseline testing**	0.371989519	0.007799815	0.005045482
**Training of TCAD ASMT**	0.002049355	0.002248726	0.002347590
**Testing of TCAD ASMT**	0.001940389	0.002230763	0.003246904

**Fig 11:**
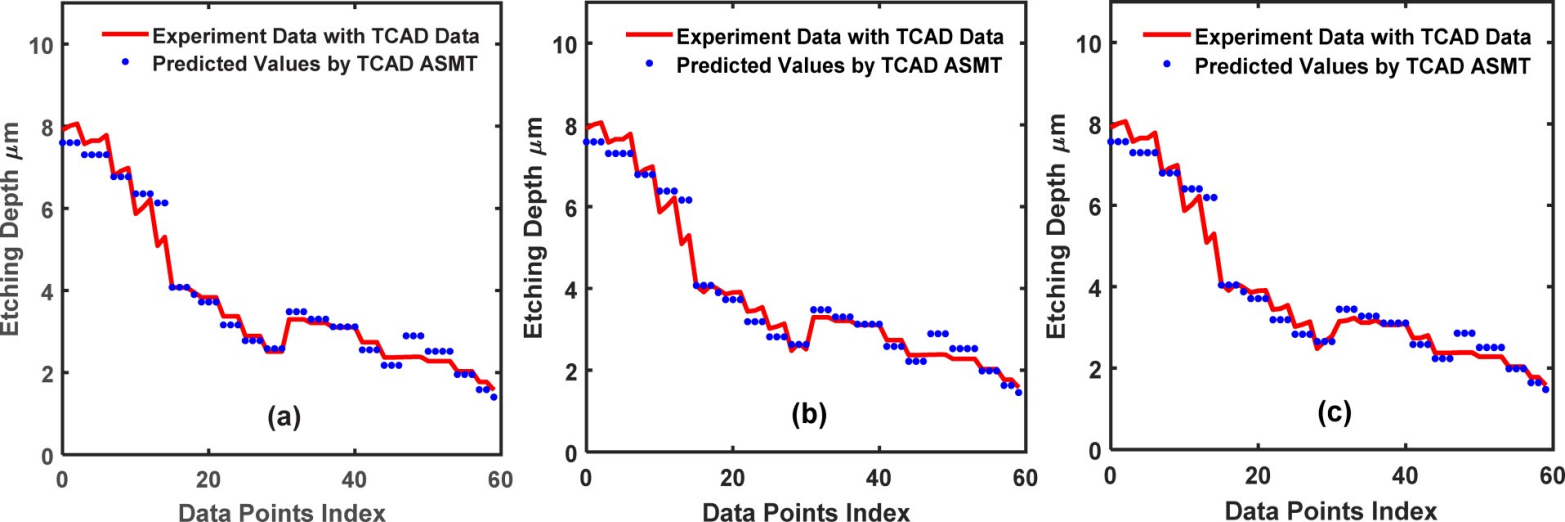
Training set fitting for the 2-input dataset using the TCAD-labeled ASMT algorithm. The partition values, 15, 30, and 45, are shown in (a), (b) and (c), respectively.

The mixed model training with data from the real experimental outputs and TCAD-labeled outputs is beneficial in terms of the accuracy of the NN, which is evident from Fig 11(A). Fig 11(A) plots the fit of NN to the dataset with mixed training, i.e., fitting to (X_Train_, Y_Train_) and (X_Extra_, Y_Extra_). It can be seen from **Table 3** that the MSE value for testing the mixed training (ASMT) model decreased to 1.9×10^−3^ from 0.37 for the baseline NN model.

**Fig 12:**
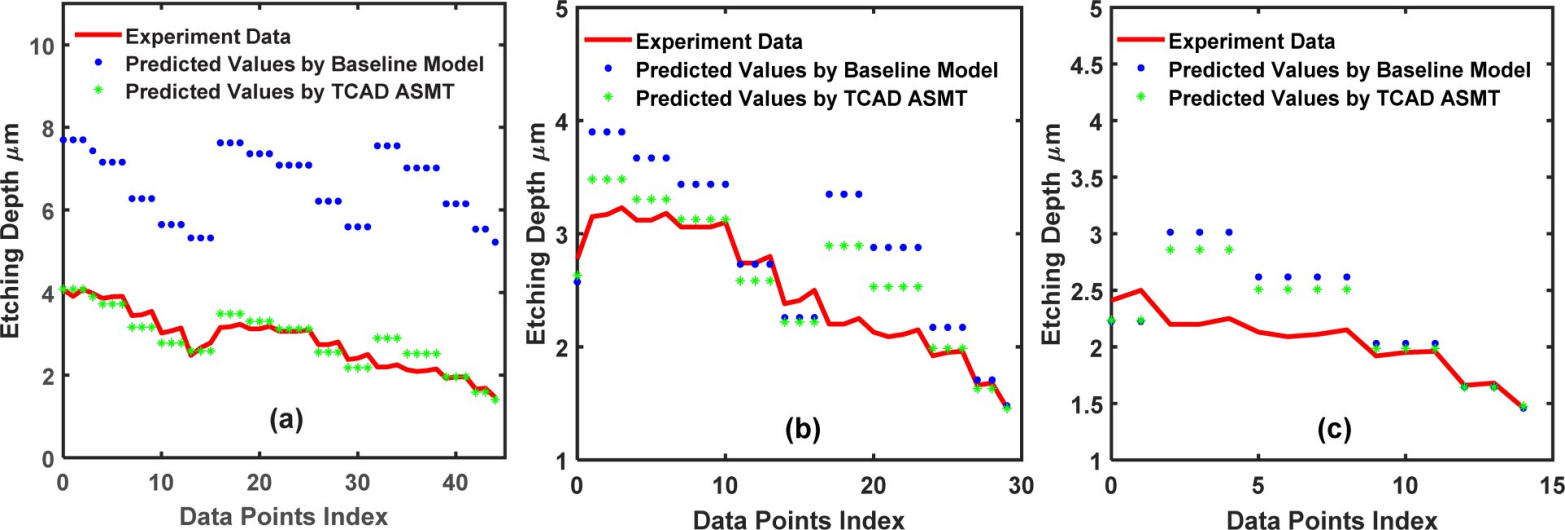
Comparison between the test set prediction using the baseline conventional NN and the TCAD-labeled ASMT algorithm for the 2-input dataset. (a), (b) and (c) show the predicted values for the partition values,15, 30, and 45, respectively.

Fig 12(A) compares the prediction accuracy for the test set of the baseline supervised NN and TCAD-labeled ASMT for *partition* = 15. The prediction of the TCAD-assisted ASMT model is far more accurate than that of baseline supervised NN due to the incorporation of TCAD to assign the partially correct output to the unlabeled data. This accuracy, in turn, helps the prediction, although the TCAD results have some inaccuracy because the TCAD outputs are still better than totally missed outputs.

In Fig 10(B) and 10(C), we show the cases of *partition* = 30 and 45, respectively. With an increased size in the training dataset, the baseline supervised NN, which fits to (X_Train_, Y_Train_), is more accurate because more data are sampled and more of the sample space information is included in the baseline neural network. A significant improvement of the TCAD-labeled ASMT over the baseline supervised NN is still clearly observed in the case of partition values of 30 and 45, as shown in Fig 12(B) and 12(C), respectively. However, the relative improvement decreased for TCAD-assisted model relative to the baseline case for these partition values (*partition* = 30 and 45) compared to *partition* = 15. Certainly, if the sampling is thorough over the entire sample space, our model will not be effective since the baseline neural network can be fully trained with the abundant training set data and the prediction on the test set will be very accurate using a conventional NN method solely. In fact, if the sampling is done at a high frequency and scattered all over the searching space, the machine learning algorithm is not necessary at all since a look-up table is sufficient to determine Y(X) for a given X. Nonetheless, such dense sampling is unattainable in many cases, such as in semiconductor manufacturing, due to the lack of a large amount of data, as mentioned earlier. Therefore, a better prediction scheme should be employed to supplement the classic NN methodology in semiconductor manufacturing.

While semiconductor TCAD has been developed for many years, the information provided by TCAD should not be abandoned completely. The motivation of this proposal is to provide a seamlessly combined method to bring together the effectiveness of both the statistical and physical methods. As far as the TCAD model is concerned, its accuracy is undoubtedly important, which in turn will affect the accuracy of TCAD-labeled ASMT learning. In general, the more sophisticated TCAD models require more computation and numerical grids. The trade-off normally needs to be made between the TCAD model accuracy and computational complexity. In fact, even a rough TCAD model can help in the prediction compared to the baseline in TCAD-labeled ASMT because the exact TCAD calculated values may deviate from the true experimental values, but the trend in the TCAD model still guides the neural network in the unsampled part of the search space. Unless the TCAD model is completely inaccurate and provides a wrong trend, which is unlikely, the incorporation of the TCAD information is always beneficial for machine learning. This aspect highlights the wide applicability of TCAD-labeled ASMT model. In most large-scale data mining problems, such as in semiconductor processing, the vast sample space strengthens the importance of the TCAD-labeled ASMT model. In our small-scale problem, where the sample space is not very large, the TCAD model can assist in the initial solution search stage where not much data have been collected or little information about the optimum place is certain. After the initial phase, the TCAD calculated values can be gradually abandoned, which can be done by replacing the TCAD calculated outputs with real sampled values or by abandoning the TCAD-labeled data directly without replacement. In either case, the TCAD model still helps in the early solution search stage since the NN constructed by TCAD-labeled ASMT learning provides useful information on the sample space based on the analytical models.

**Fig 13:**
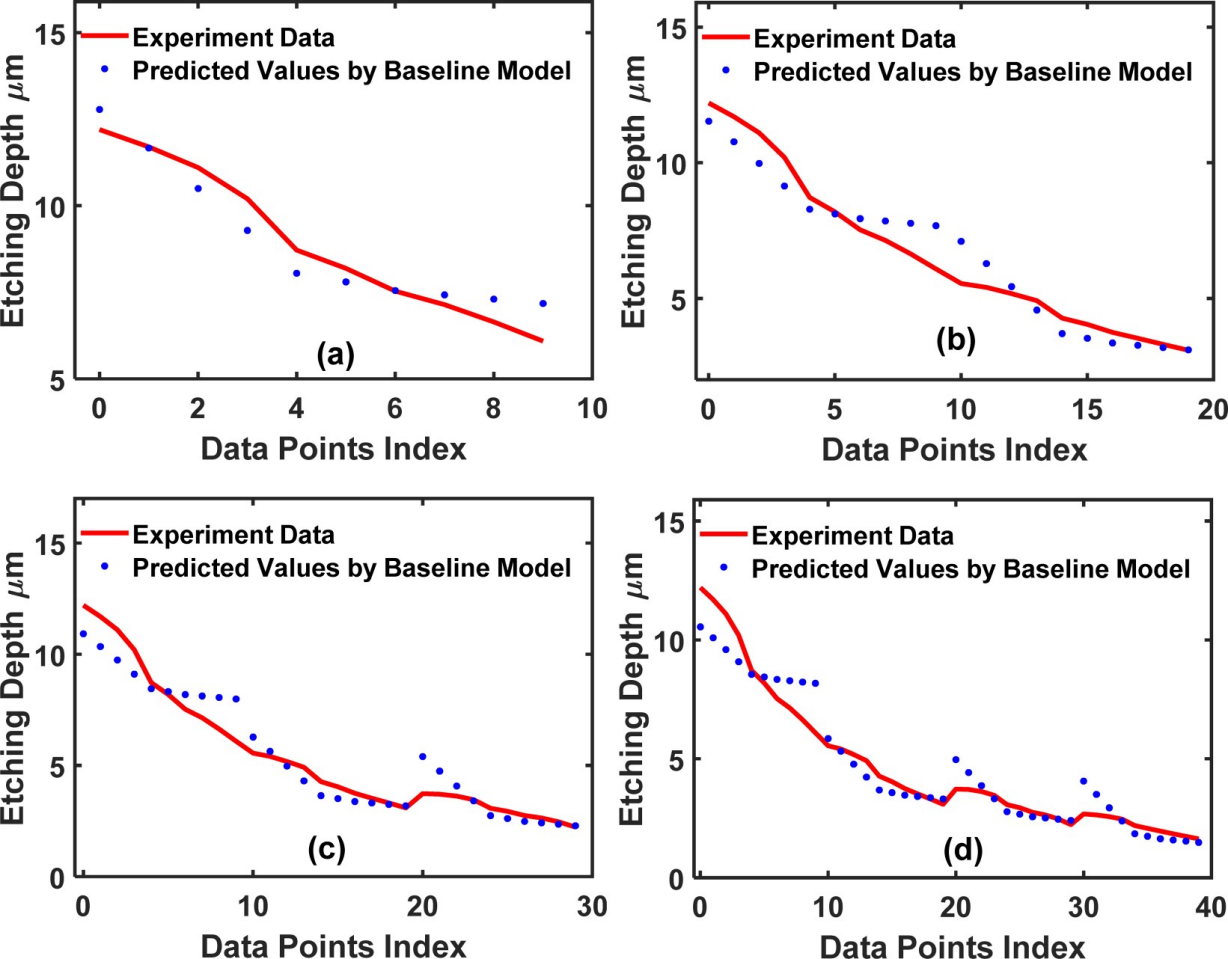
Training set fitting for the 5-input dataset using the baseline conventional NN. The partition values, 10, 20, 30 and 40, are shown in (a), (b), (c) and (d), respectively.

**Fig 14:**
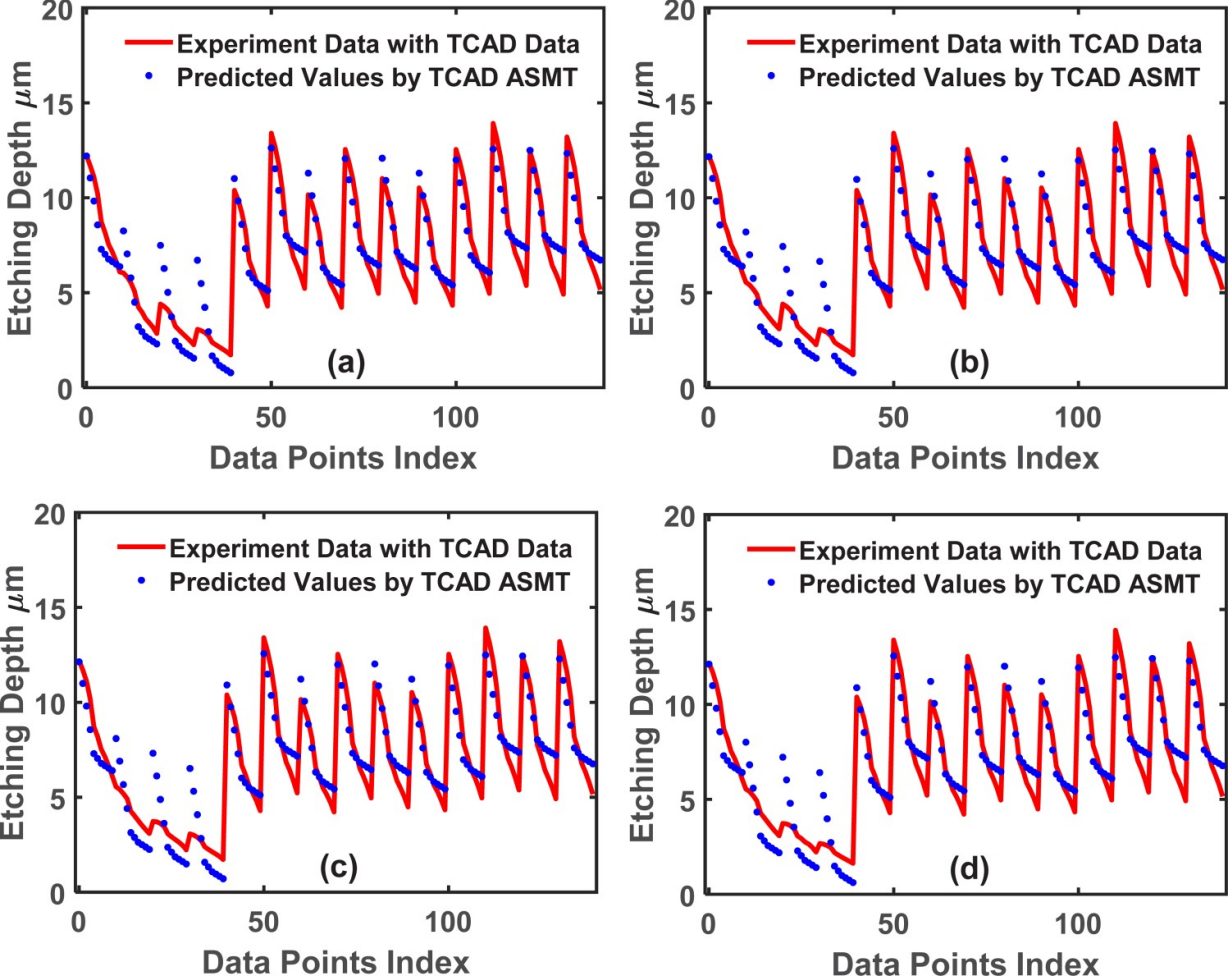
Training set fitting for the 5-input dataset using the TCAD-labeled ASMT algorithm. The partition values, 10, 20, 30, and 40, are shown in (a), (b), (c)and (d), respectively.

**Fig 15:**
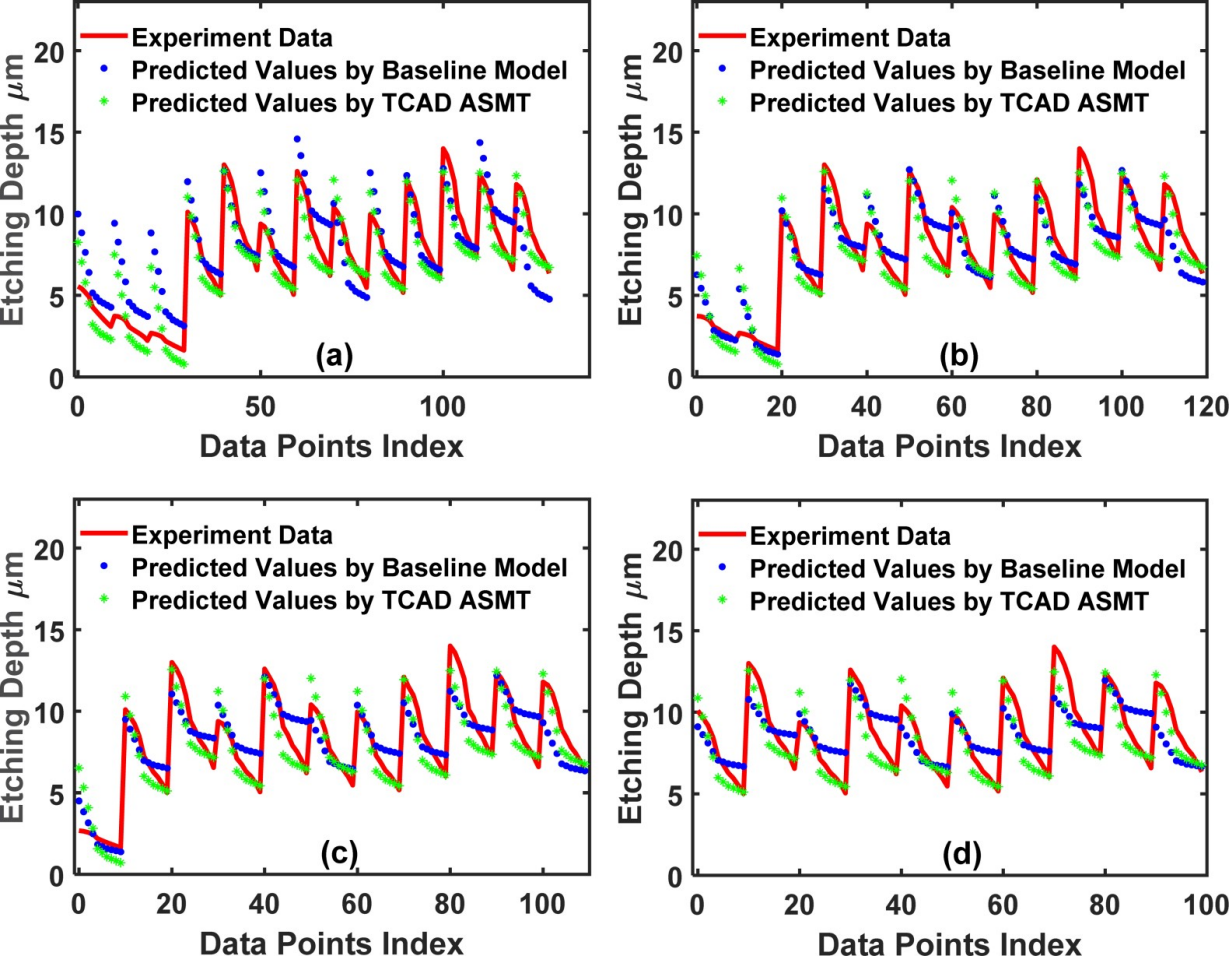
Comparison between the test set prediction using the baseline conventional NN and the TCAD-labeled ASMT algorithm for the 5-input dataset. (a), (b), (c) and (d) show the predicted values for the partition values, 10, 20, 30 and 40, respectively.

To demonstrate the wide applicability of TCAD-labeled ASMT model, we use a different dataset to repeat the algorithm. This dataset is more complex and has 5 input features: the trench width (*W*), etching time (*t*), etching pressure (*P*), etching SF6 flow (*F*) and ICP RF main etch (*IF*). In the DRIE Bosch process, these are all critical tuning parameters to attain an optimal etched trench. Fig 13 plots the training set fitted by (X_Train_, Y_Train_), and Fig 14 plots the training set fitted by (X_Train_, Y_Train_) and (X_Extra_, Y_Extra_). Fig 15 shows a comparison of the test set prediction results of the baseline NN and TCAD-labeled ASMT model. Similar to the previous dataset calculations, we have partitions of 10, 20, 30, and 40. Significant improvement is evident in Fig 15, and the corresponding MSE values are presented in **Table 4**.

**Table 4:** MSE values of the training and testing of the 5-input dataset for the TCAD-labeled ASMT algorithm.

Partition value	10	20	30	40
**Training of baseline**	0.002578363	0.004218990	0.004465175	0.004173959
**Testing of baseline**	0.024082398	0.011346209	0.012909928	0.015625874
**Training of TCAD ASMT**	0.007453523	0.007679670	0.008003452	0.008165518
**Testing of TCAD ASMT**	0.009290367	0.009047419	0.008231916	0.007124938

From the real fabrication and numerical experiments demonstrated through a DRIE BOSCH process, it is shown that the TCAD-labeled ASMT is a more efficient algorithm reference to conventional neural networks. In fact, the physical models have already been developed in the early days of many fields, including geography, astrophysics, climate, and biology. While machine learning has become more prominent in these fields in recent years, we suggest that the incorporation of well-developed physical and analytical modeling is always beneficial.

## 4. Conclusions

In this work, we use a TCAD-labeled analytics-statistics mixed training (ASMT) model and apply it to an ARDE problem. The effectiveness of machine learning in intelligent semiconductor manufacturing can become more pronounced by incorporating TCAD analytical models in terms of cost-efficiency, prediction accuracy, and reduced trial-and-error cycles. The TCAD-labeled ASMT is particularly effective when there is under sampling in the data mining process, which inevitably happens due to unbounded and vast searching space. This case is the typical scenario encountered in semiconductor processing since advanced IC technologies require the optimization of hundreds of process steps with thousands or more input parameters. Even when using a straightforward aerodynamic model for the DRIE Bosch process, TCAD-labeled ASMT machine learning model demonstrates a significant reduction in the MSE values relative to that of the conventional neural network methodology without the TCAD labels. The underlying mathematical reasoning for the improvement is understood as the partially correct TCAD labels assisting in the training procedure for the neural network weights, and as a result, the statistical induction based on machine learning and analytical deduction based on fluid dynamics and chemical process dynamics jointly improve the prediction accuracy. Additionally, our TCAD-labeled ASMT algorithm can easily fit the semisupervised learning (SSL) framework. A better, more sophisticated analytical model can further improve the effectiveness of our methodology, while even simple models with correct trends can already have a pronounced effect because they at least supplement the unlabeled data that contain no relevant information at all. With over 30 years of TCAD model development in the field of semiconductor physics, we suggest the combined TCAD and machine learning approach, which is more effective, saves CPU runtime manufacturing costs, and does not waste the prior contributions of the semiconductor device and process modeling.
